# Toll-Like Receptor 2 Is a Regulator of Circadian Active and Inactive State Consolidation in C57BL/6 Mice

**DOI:** 10.3389/fnagi.2017.00219

**Published:** 2017-07-14

**Authors:** Nicholas W. DeKorver, Tammy R. Chaudoin, Stephen J. Bonasera

**Affiliations:** Division of Geriatrics, Department of Internal Medicine, Durham Research Center II, University of Nebraska Medical Center Omaha, NE, United States

**Keywords:** active/inactive state consolidation, sleep fragmentation, Tlr2 knockout mouse, home cage behavior, circadian behavior, movement

## Abstract

Regulatory systems required to maintain behavioral arousal remain incompletely understood. We describe a previously unappreciated role that toll-like receptor 2 (Tlr2, a membrane bound pattern recognition receptor that recognizes specific bacterial, viral, and fungal peptides), contributes toward regulation of behavioral arousal. In 4–4.5 month old mice with constitutive loss of Tlr2 function (Tlr2^−/−^ mice), we note a marked consolidation in the circadian pattern of both active and inactive states. Specifically, Tlr2^−/−^ mice demonstrated significantly fewer but longer duration active states during the circadian dark cycle, and significantly fewer but longer duration inactive states during the circadian light cycle. Tlr2^−/−^ mice also consumed less food and water, and moved less during the circadian light cycle. Analysis of circadian rhythms further suggested that Tlr2^−/−^ mice demonstrated less day-to-day variability in feeding, drinking, and movement behaviors. Reevaluation of this same mouse cohort at age 8–8.5 months revealed a clear blunting of these differences. However, Tlr2^−/−^ mice were still noted to have fewer short-duration active states during the circadian dark cycle, and continued to demonstrate significantly less day-to-day variability in feeding, drinking, and movement behaviors. These results suggest that Tlr2 function may have a role in promoting transitions between active and inactive states. Prior studies have demonstrated that Tlr2 regulates sickness behaviors including hypophagia, hyperthermia, and decreased activity. Our work suggests that Tlr2 function also evokes behavioral fragmentation, another aspect of sickness behavior and a clinically significant problem of older adults.

## Introduction

Older adults often experience sleep fragmentation, a geriatric syndrome characterized by multiple awakenings and arousals that disrupt normal sleep architecture (Huang et al., 2002). Sleep fragmentation is associated with significant clinical outcomes, including cognitive impairment (Oosterman et al., 2009; Lim et al., 2012), falls (Stone et al., 2008), decreased patient quality of life (Hidalgo et al., 2007), and social isolation (Kurina et al., 2011). In adults older than 65, insomnia and sleep disordered breathing (usually from obstructive sleep apnea) account for 60 and 40% of sleep fragmentation complaints, respectively (Birath and Martin, 2007). CNS aging (Huang et al., 2002; Bliwise et al., 2009; Lim et al., 2015) and CNS neurodegeneration (Vitiello et al., 1990; Porter et al., 2008; Lim et al., 2016, among others) both contribute significantly to insomnia.

Sleep is a highly complex physiological process organized over multiple sites within the CNS. The hypothalamic lateral nucleus (LH) is a critical region for the generation and regulation of sleep behaviors. The LH contains a discrete neuronal population that expresses orexin, a 33/28 peptide hormone (orexin A/B, respectively) first described for its involvement in narcolepsy, an acquired sleep disorder (Lin et al., 1999). Mice genetically engineered to lack prepro-orexin, orexin receptor-2 (Chemelli et al., 1999), or orexin (Willie et al., 2003) exhibit severe sleep fragmentation. Electrophysiological studies demonstrate that resting membrane voltages in orexinergic neurons are intrinsically depolarized (Eggermann et al., 2003), making them exquisitely sensitive to excitatory synaptic input (Acuna-Goycolea et al., 2004; Henny and Jones, 2006; Alberto and Hirasawa, 2010). Orexinergic neurons share reciprocal inhibitory projections with LH neurons expressing melanin-concentrating hormone (MCH). Data obtained from optogenetic approaches strongly supports the concept that under basal conditions (e.g., without sleep pressure; Carter et al., 2009), joint activity of orexinergic and MCH neurons constitutes a biological “switch” that toggles between wake (active) and sleep (inactive) states (Adamantidis et al., 2007; Konadhode et al., 2013).

Molecules regulating orexinergic neuron firing properties may therefore play an important role in the development of age-associated sleep fragmentation. For example, hypothalamic toll-like receptors have been strongly linked to “sickness behavior,” a set of coordinated behaviors (including increased sleepiness, anorexia, and lethargy) that focus metabolic resources toward fighting infections (Farzi et al., 2015; Reis et al., 2015). One member of this family, toll-like receptor 2 (Tlr2), has a prominent role in organizing sickness behavior (Hübschle et al., 2006; Jin et al., 2016). Tlr2 is an intrinsic membrane protein containing multiple extracellular tandem leucine-rich-repeat motifs that fold into a characteristic horseshoe shape (Jin and Lee, 2008). Tlr2 forms heterodimers with similarly shaped partners such as toll-like receptor 1 (Tlr1), or toll-like receptor 6 (Tlr6). Tlr2 also forms homodimers with itself (Farhat et al., 2008; van Bergenhenegouwen et al., 2013). These dimers, when organized into larger structures containing Cd14 and Cd36, recognize extracellular diacylated and triacylated lipopeptide ligands (Yang et al., 1998). Intracellular signaling cascades initiated by Tlr2 are quite complex (Oda and Kitano, 2006), but may be summarized by (1) Tlr2 ligand binding recruiting the adaptor protein MyD88, (2) MyD88 activation of pathways that phosphorylate IκB and MAPK, causing (3) nuclear translocation of NFκB (Akira and Sato, 2003) and AP1 (Plotnikov et al., 2011). In neurons, NFκB signaling regulates axon growth (Kaltschmidt and Kaltschmidt, 2009), dendrite arborization (Gutierrez et al., 2005), synapse formation and plasticity (Freudenthal et al., 2004; Boersma et al., 2011), and behavior (Meffert et al., 2003). NFκB is also the major transcriptional regulator of Tlr2, as well as other cytokines (IL-1β, IL-6, TNF-α, *etc*.) implicated in sickness behavior. Finally, there is ample data demonstrating that Tlr2 is expressed in CNS cell types and regions relevant to sleep/wake cycle regulation. Most cell types within the brain, including microglia, astrocytes, neurons, and oligodendrocytes, have been shown to express Tlr2 (Bsibsi et al., 2002; Tang et al., 2007). Orexinergic neurons strongly express both Tlr2 transcript and protein (Dalal et al., 2013). Additionally, hypothalamic expression of Hsp70 occurs under a variety of stressor conditions (Kageyama et al., 2000; Suzuki et al., 2003), and may play a role in Tlr2 signaling pathways (Borges et al., 2012). In summary, there is much evidence that Tlr2-mediated orexinergic neuron regulation plays a role in expression of sickness behaviors.

In this manuscript, we present data from home cage behavioral monitoring experiments showing that constitutive loss of Tlr2 has a previously unappreciated role in consolidating shorter periods of activity or inactivity in C57BL/6 mice into longer duration active or inactive states. In 4–4.5 months old mice, this consolidation has dramatic effects on overall measures of feeding, drinking, and movement, particularly during the light cycle. Older (8–8.5 months) Tlr2^−/−^ mice continue to show both active and inactive state consolidation, with no genotypic effects on feeding, drinking, and movement.

## Methods

### Mice

Tlr2^tm1Aki^ homozygous mutant mice (engineered with a constitutive mutation deleting a transmembrane domain and protein cytoplasmic tail; see Takeuchi et al., 1999) were obtained from a colleague's colony and triad-mated to create cohorts of mutant mice. We confirmed presence of this genetic lesion by PCR of DNA obtained from tail biopsy of putative Tlr2^−/−^ mice. WT male and female (C57BL/6) mice were obtained from Jackson labs and triad-mated to create a WT colony. Since Tlr1, Tlr6, and Tlr10 do not form homodimers, Tlr2^−/−^ mice cannot transduce intracellular signals evoked by exogenous or endogenous Tlr2 ligands (Zähringer et al., 2008; Erridge, 2010), and do not have a dominant negative phenotype evoked from aberrant Tlr1, Tlr6, or Tlr10 signaling. Prior to behavioral testing, mice were housed in the UNMC vivarium at a density of ≤5 per cage in a microisolator system (Lab Products Inc., Seaford DE), provided with chow (Envigo Teklad #7012) and water *ad libitum*, given environmental enrichment (Crinkle Paper Pouches, WF Fisher), and maintained on a 12:12 circadian lighting cycle (lights on 0600 CST). Vivarium temperatures ranged between 20 and 23°C. Mice were separated by sex at weaning. All studies were performed in full concordance with both institutional and federal regulations regarding animal care and use. The protocol was approved by the UNMC Institutional Animal Care and Use Committee (IACUC).

### Body mass composition determination

We performed a longitudinal assessment using dual emission x-ray absorptiometry (DEXA) for *in vivo* estimates of mouse adiposity. We evaluated one cohort of male WT (*n* = 8) and male Tlr2^−/−^ (*n* = 8) mice at age of 4–4.5 and 8–8.5 months. One mouse was lost by attrition from the 8–8.5 months old WT cohort. Before data collection, all instrumentation was calibrated to a phantom approximating mouse body composition characteristics. Briefly, mice were lightly anesthetized with isoflurane, and imaged (Piximus I, GE Lunar). Measures of body mass composition, including bone mass density (BMD), bone mineral content (BMC), bone area (BArea), tissue area (TArea), ratio of soft tissue attenuation (R_ST_), total tissue mass (TTM), and percent adiposity (% fat) were calculated from images using vendor provided software (Piximus 2.10). Differences in cohort DEXA and body weight values were determined by unpaired two-sided Student *t*-tests, with Bonferroni correction of the critical p to control the false positive rate.

### Metabolic rate determination

We performed a longitudinal assessment of mouse metabolic parameters using indirect calorimetry. Mouse cohorts as above. Briefly, mice were fasted overnight prior to metabolic assay. Animals were placed individually in a calorimetry chamber (8 total; Oxymax, Columbus Instruments), and tested between 12:00 and 17:00 for 1 day. Measures of metabolic rate, including maximum oxygen uptake ($\dot{\text{V}}$O_2_), global oxygen delivery (DO_2_), oxygen output (O_2_out), maximum CO_2_ production, ($\dot{\text{V}}$CO_2_), global CO_2_ removal (DCO_2_), CO_2_ output (CO_2_out), and heat generated were calculated using vendor provided software (Oxymax for Windows 4.49). These metabolic parameters were adjusted for mouse adiposity per ANCOVA (Tschöp et al., 2012). Full details regarding our system components and operation are provided in Bonasera et al. (2017).

### Home cage behavioral monitoring

We performed a longitudinal assessment of mouse home cage behaviors of feeding, drinking, movement, and circadian rhythm. Mouse cohorts as above. Briefly, mice were placed in a custom-designed home cage monitoring arena (32 total) with *ad libitum* access to milled chow (#5058, PicoLab) and water. This system provides high spatial (within 0.5 cm) and temporal (within 1 ms) precision of all mouse behaviors. Mice are habituated to the home cage environment for 5 days before start of data collection, which then proceeds for at least 15 days (to ensure collection of at least 14 days of data for each mouse). The system collects data at all times (except brief intervals every 3–4 days for replacement of mouse food and water supplies). After the mouse is introduced to the home cage, it is not handled until the end of the experiment. Since mouse handling is a well-appreciated stressor known to alter many behaviors (Hurst and West, 2010), we thus capture mouse home cage behaviors without imposing significant external stressors. Behavioral data obtained from this system describing C57BL/6 mice are highly similar regardless of system location or investigator (Tecott, Goulding, personal communication). Data passing automated quality control procedures are then classified to determine mouse active/inactive states, mouse intake (of food and water) bouts, and mouse movement (locomotion and movement-in-place) bouts. Following classification, we determine up to 665 distinct measures of mouse feeding, drinking, movement, and circadian activity. We employ false discovery rate (FDR) statistics to minimize family-wise error rates; behaviors found significant in this manner are subjected to further analysis. Full details regarding our system hardware and software characteristics have been published (Goulding et al., 2008; Parkison et al., 2012; Bonasera et al., 2017).

We examined circadian periodicities using Lomb-Scargle analysis (Lomb, 1976; Scargle, 1982); this method detects multiple periodicities within a time series and has been validated in the setting of incompletely sampled data streams. Feeding, drinking, and movement data were binned into 6-min epochs, and significant periodicities (up to 60 h duration) were calculated using an implementation described by Van Dongen et al. (1999) and coded in MATLAB 2011b (MathWorks, Natick MA). We examined patterns of active state onset and duration using the comparison clustering algorithm described by Goulding et al. (2008) and coded in MATLAB 2011b. Briefly, active state onset and duration data from both the WT and Tlr2^−/−^ groups are combined. Between 2 and 50 clusters were fit to this dataset using bivariate normal distributions. We then test the null hypothesis that WT and Tlr2^−/−^ mice are equally represented in a given cluster by calculating a χ^2^ statistic comparing the observed to predicted number of data points that WT and Tlr2^−/−^ contribute to this cluster. The overall difference between WT and Tlr2^−/−^ mice active state patterns is thus represented by the sum of χ^2^ values over all clusters.

The major limitations of our home cage behavioral monitoring approach include brief epochs of data loss secondary to blocked photobeams (e.g., chow pile, or resting mouse close enough to encroach on the beam radius), licker malfunction (in particular, drips), mouse weight gain/loss during the experiment, and excessive rearing along the cage wall (both altering reported movement distances). All of these issues are identified during our data quality control phase, and removed from the dataset at that time. Since these issues occur infrequently and at random times, there are no statistical problems introduced by removing this small percentage of collected data during the quality control phase.

## Results

### False discovery rate (FDR) identifies 73 differentially expressed behaviors between 4 and 4.5 months old WT and Tlr2^−/−^ mice, but no behaviors differentially expressed between the same mice at 8–8.5 months old

**Figure 1:**
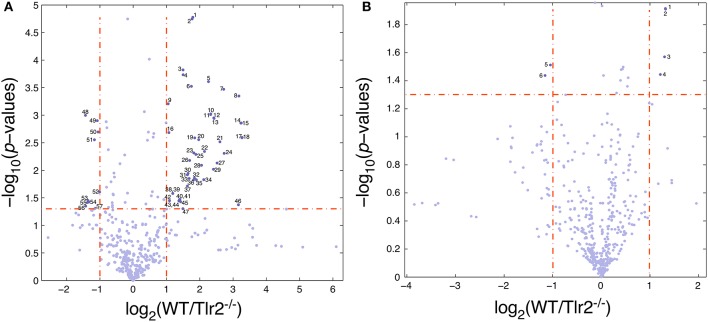
Volcano plot of differentially expressed behaviors between wildtype and Tlr2^−/−^ cohorts **(A)**. 4–4.5 months old cohort. Generally, Tlr2^−/−^ mice demonstrated fewer behaviors overall during the circadian light cycle, and had altered active/inactive state properties. Dashed vertical lines depict boundaries for two-fold decreases (left) and increases (right) in behavior (WT compared to Tlr2^−/−^); dashed horizontal line depicts behavioral significance *p* < 0.05. Behaviors between the left and right dashed vertical lines show no significant differences between WT and Tlr2^−/−^ mice. Descriptions of identified behaviors are provided in columns A-C of Supplemental Table 1. Abbreviations forming behavior names are as follows: AFL: activity-feeding-licking overall averages; TBA: time budget analysis; SA: active/inactive state analysis; BA: intake/movement bout analysis; BD: bout dominance analysis; DCLC: behaviors aggregated across both dark and light cycles; DC: behaviors aggregated over dark cycle; LC: behaviors aggregated over light cycle; PE: photobeam event (feeding); LE: lickometer event (drinking), ME: movement event; AP: mouse active phase; IP: mouse inactive phase. Identified behaviors (per column A) are: (1) BA_ME_DCLC_Go_LC_ActProb, (2) TBA_ DCLC_MeanPerAct_LC, (3) TBA_DCLC_MeanPerStop_LC, (4) BA_ME_DCLC_Other_ LC_BoutTotDur_ms, (5) SA_IP_NumActStates_EatDrink, (6) BA_ME_DCLC_Other_LC_ BoutTotMove_cm, (7) SA_NumActStates_SmallEatLargeDrink, (8) SA_IP_NumActStates_ SmallEatLargeDrink, (9) BA_ME_DCLC_Stop_LC_BoutTotMove_cm, (10) BA_PE_DCLC_ Small_LC_BoutRate_onperms, (11) BA_PE_DCLC_Small_LC_BoutNumber, (12) TBA_ DCLC_MeanPerFeed_LC, (13) BA_PE_DCLC_Large_LC_MeanToralBoutDur_ms, (14) BA_ PE_DCLC_Large_LC_BoutRate_onperms, (15) BA_PE_DCLC_Large_LC_BoutNumber, (16) AFL_avg_LC_Move_m, (17) BA_LE_DCLC_Large_LC_BoutRate_onperms, (18) BA_LE_ DCLC_Large_LC_BoutNumber, (19) SA_PerActStates_SmallEatLargeDrink, (20) BA_PE_DCLC_Large_LC_MeanTotalBoutIntake_mg, (21) SA_AP_PerActiveStates_ SmallEatSmallDrink, (22) BA_PE_DCLC_Small_LC_MeanTotalBoutIntake_mg, (23) AFL_avg_LC_Chow_gkg, (24) SA_AP_NumActiveStates_SmallEatSmallDrink, (25) BA_LE_ DCLC_Large_LC_BoutRate_onperactms, (26) AFL_avg_LC_Liquid_g (27) SA_AP_ NumActStates_NoEatNoDrink, (28) SA_AP_NumActStates_SmallEatLargeDrink, (29) BA_PE_DCLC_Small_LC_MeanTotalBoutDur_ms, (30) BA_ME_DCLC_Other_LC_BoutRate_onperms, (31) BA_ME_DCLC_Other_LC_BoutMeanNumber, (32) SA_ NumActStates_SmallEatSmallDrink, (33) BA_LE_DCLC_Small_LC_BoutProbability, (34) SA_AP_PerActStates_NoEatNoDrink, (35) TBA_DCLC_MeanPerStopAtOther_LC, (36) SA_IP_NumActStates_LargeEatLargeDrink, (37) SA_AP_PerActStates_SmallEatLargeDrink, (38) BA_PE_DCLC_Large_BoutRate_onperms, (39) BA_PE_DCLC_Large_BoutNumber, (40) BA_ME_DCLC_Stop_LC_BoutRate_onperms, (41) BA_ME_DCLC_Go_LC_BoutRate_onperms, (42) SA_PerActStates_SmallEatSmallDrink, (43) BA_ME_DCLC_Stop_LC_ BoutMeanNumber, (44) BA_ME_DCLC_Go_LC_BoutMeanNumber, (45) BA_ME_DCLC_Go_LC_BoutTotMove_cm, (46) BA_LE_DCLC_Small_LC_BoutRate_onperactms, (47) SA_ IP_NumActStates_SmallEatSmallDrink, (48) SA_DCLC_LC_MeanInactMove_cm, (49) BA_ LE_DCLC_Large_BoutSlopeIntensity_mgs, (50) BA_LE_DCLC_Large_DC_ BoutSlopeIntensity_mgs, (51) SA_DCLC_LC_MeanInactDur_ms, (52) TBA_DCLC_ MeanPerActShortStopAtHomeBase_LC, (53) SA_DCLC_MeanInactMove_cm, (54) BA_PE_ DCLC_DC_BoutMeanDur_ms, (55) BA_PE_DCLC_Small_DC_BoutMeanSize_evtdur, (56) BA_PE_DCLC_Small_DC_BoutMeanSize_mg, (57) BA_PE_DCLC_Small_BoutMeanDur_ms. **(B)**. 8–8.5 months old cohort. Behaviors significantly differing between WT and Tlr2^−/−^ mice depicted per **(A)**. Supplemental Table 1 provides descriptions for each of these behaviors. Identified behaviors are (1) BD_Chow_BtSizeRsq, (2) BD_Chow_BtSizePerRSq, (3) SA_NumActStates_SmallEatLargeDrink, (4) SA_AP_NumActStates_SmallEatLargeDrink, (5) BA_ PE_DCLC_Small_TotalDur_ms, (6) BA_PE_ DCLC_Small_PerCumIntake.

Most of the genotypic differences between the WT and Tlr2^−/−^ 4–4.5 months old cohorts involve behaviors occurring during the circadian light cycle (46 of the 73 total, Supplemental Table 1). Tlr2^−/−^ mice showed significantly less light cycle movement, feeding, and drinking, and budgeted significantly less time to perform these behaviors. Bout metrics for light cycle intake/movement onsets, intake/movement durations, per bout intake/movement, bout intake/movement intensities, and bout intake rates/movement speeds all show large genotypic differences as a consequence of the overall paucity of Tlr2^−/−^ light cycle behaviors. Inactive and active state properties (discussed in next section) were generally characterized by Tlr2^−/−^ mice having fewer daily active/inactive states, lower active/inactive state transition rates, but longer active/inactive state durations; all consistent with the concept that Tlr2^−/−^ mice have consolidated active and inactive states. Figure 1A is a volcano plot that displays two-fold changes in specific behaviors between WT and Tlr2^−/−^ above the horizontal dashed line; metrics where WT were significantly greater than Tlr2^−/−^ are depicted right of the right vertical dashed line, metrics where WT were significantly less than Tlr2^−/−^ are depicted left of the left vertical dashed line. In this figure, 38 of the 57 behaviors identified as having genotypic differences were observed during the light cycle.

Interestingly, at 8–8.5 months of age, these same mouse cohorts demonstrated far fewer genotypic differences in behavior. No differences were appreciated by FDR analysis (*p* < 0.05, Supplemental Table 1); significant differences in 6 behaviors were noted by volcano plot (Figure 1B).

### 4–4.5 months old Tlr2^−/−^ mutant mice demonstrate greater active and inactive state stability

**Figure 2:**
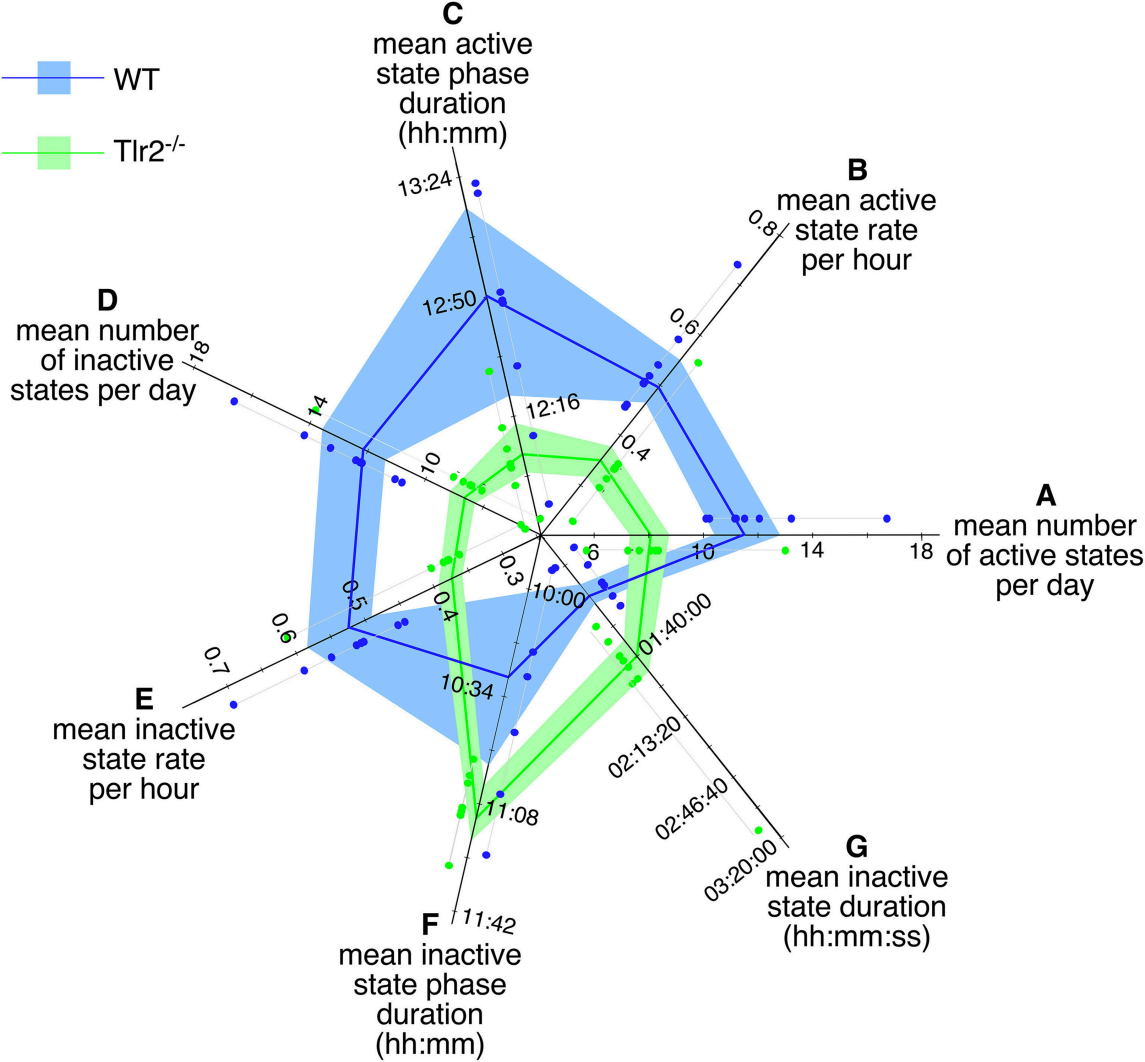
4–4.5 months old Tlr2^−/−^ mice have fewer, more consolidated active and inactive states throughout the day. Polygon plot of significant daily active and inactive state properties. Light blue region spans the first through third data quartiles for all WT mice; the dark blue line depicts property median values for WT. Similarly, light green region spans the first through third data quartiles for all Tlr2^−/−^ mice; the dark green line depicts property median values for all Tlr2^−/−^. Thin gray lines group data points for each genotype/property combination; these values are offset from their corresponding axis for clarity **(A)**. Number of daily active states **(B)**. Daily active state rate (per hour) **(C)**. Daily active state phase duration **(D)**. Daily number of inactive states **(E)**. Daily inactive state rate (per hour) **(F)**. Daily inactive state phase duration **(G)**. Median duration for a single inactive state. Note that axis origins do not start at 0.

As mentioned above, most of the behaviors phenotypically differing between young WT and Tlr2^−/−^ cohorts reflected significant changes in how these two genotypes regulate active states. We therefore decided to examine mouse active and inactive state properties in greater detail. Over the combined dark and light cycles, Tlr2^−/−^ mice had fewer active states per day (Figure 2A, Table 1), fewer active states per hour (Figure 2B), and a shorter active phase (Figure 2C) compared to WT mice. Active state durations trended to be longer in Tlr2^−/−^ mice, but this finding did not achieve statistical significance. Similarly, over the combined dark and light cycles Tlr2^−/−^ mice had fewer inactive states per day (Figure 2D), fewer inactive states per hour (Figure 2E), a longer inactive phase (Figure 2F), and longer duration inactive states (Figure 2G) compared to WT. These findings are consistent with Tlr2^−/−^ mice experiencing fewer transitions between active and inactive states throughout the circadian day, and thus having increased state stability.

**Table 1:** Comparison of overall active and inactive state properties between 4 and 4.5 months old WT and Tlr2^−/−^mice.

**Behavior**	**WT (mean ± *sd*)**	**Tlr2^−/−^ (mean ± *sd*)**	***P***
Number daily active states	12.2 ± 2.1	8.3 ± 2.3	<0.005
Active states per hour	0.52 ± 0.09	0.36 ± 0.1	<0.005
Active phase duration (s) (hh:mm:ss)	45,652 ± 1,834	43,476 ± 813	<0.0126
	12:40:53 ± 00:30:43	12:04:36 ± 00:13:33	
Number of daily inactive states	12.7 ± 1.9	9.1 ± 2.4	<0.006
Inactive states per hour	0.55 ± 0.08	0.39 ± 0.1	<0.007
Inactive phase duration (s)	37,904 ± 1,874	40,159 ± 648	<0.01
(hh:mm:ss)	10:31:44 ± 00:31:14	11:09:19 ± 00:10:48	
Inactive state duration (min)	64.9 ± 9.9	108.7 ± 35	<0.004

**Figure 3:**
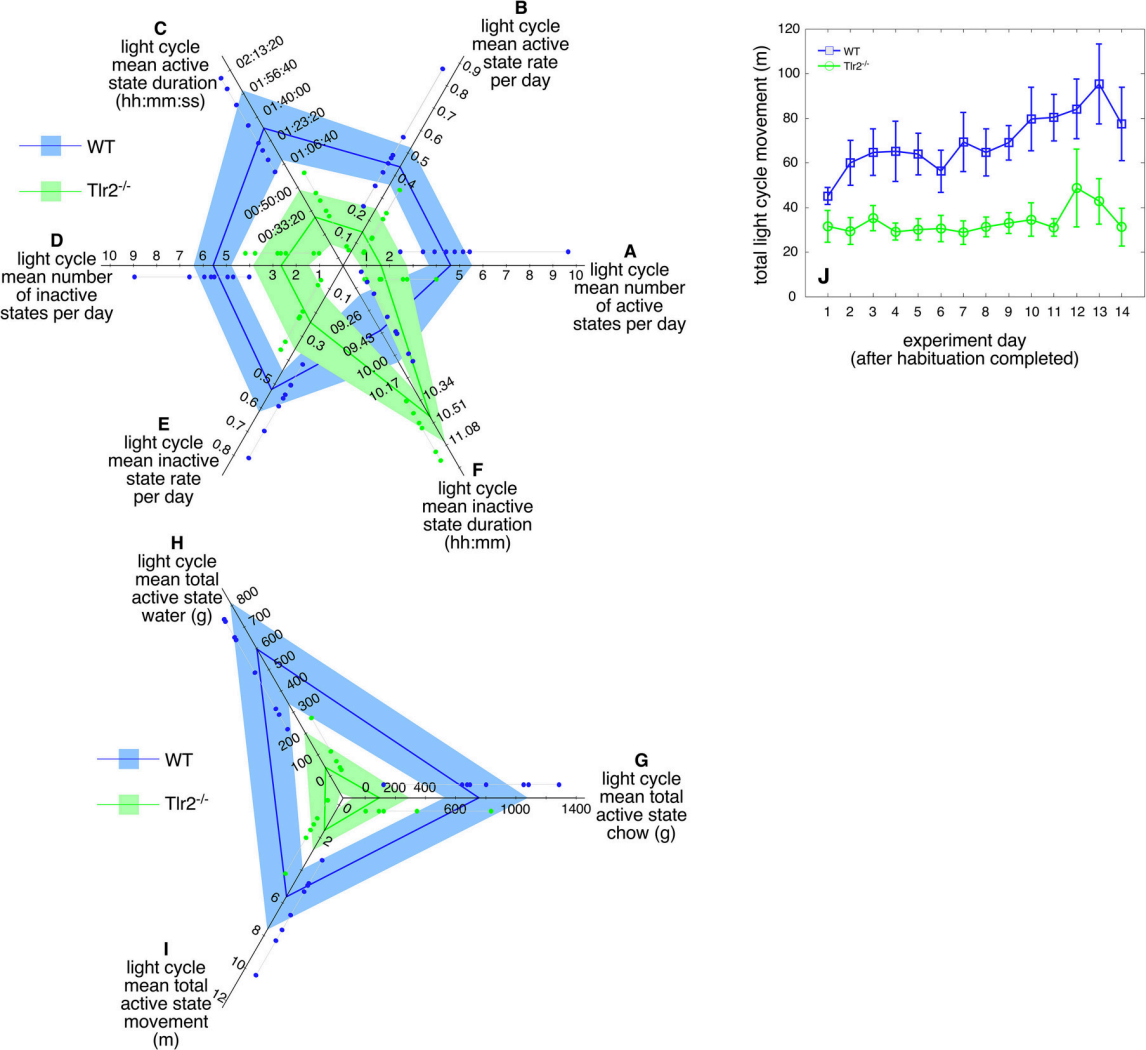
4–4.5 months old Tlr2^−/−^ mice demonstrate dramatic behavioral consolidation during the circadian light cycle. Polygon plots of significant light cycle active and inactive state properties **(A)**. Number of circadian light cycle active states. Light blue region spans the first through third data quartiles for all WT mice; the dark blue line depicts property median values for WT. Similarly, light green region spans the first through third data quartiles for all Tlr2^−/−^ mice; the dark green line depicts property median values for all Tlr2^−/−^. Thin gray lines group data points for each genotype/property combination; these values are offset from their corresponding axis for clarity **(B)**. Circadian light cycle active state rate **(C)**. Circadian light cycle active state duration **(D)**. Circadian light cycle number of inactive states **(E)**. Circadian light cycle inactive state rate **(F)**. Circadian light cycle inactive state duration **(G)**. Circadian light cycle chow consumption **(H)**. Circadian light cycle water consumption **(I)**. Circadian light cycle movement **(J)**. Total daily light cycle movement.

This altered active/inactive state transition frequency is particularly marked during the circadian light cycle (Table 2). Compared to WT, Tlr2^−/−^ mice expressed fewer active states (Figure 3A), had a lower active state rate (Figure 3B), and a shorter active state duration (Figure 3C) during the circadian light cycle. Similarly, Tlr2^−/−^ mice had fewer inactive states (Figure 3D), a lower inactive state rate (Figure 3E), and a longer inactive state duration (Figure 3F) during the circadian light cycle. These values suggest that WT mice spend 13% of the light cycle in an active state, while Tlr2^−/−^ mice spend 4% of the light cycle in an active state. Not surprisingly, decreased light cycle activity duration in Tlr2^−/−^ mice was accompanied by decreased feeding, drinking, and movement compared to WT (Figures 3G–J respectively). Tlr2^−/−^ mice clearly experience fewer transitions between active/inactive states during the circadian light cycle compared to WT.

**Table 2:** Comparison of light cycle (LC) overall active and inactive state properties between 4 and 4.5 months old WT and Tlr2^−/−^mice.

**Behavior**	**WT (mean ± *sd*)**	**Tlr2^−/−^(mean ± *sd*)**	***P***
Number light cycle active states	4.9 ± 2.1	2.1 ± 1.1	<0.007
Light cycle active states per hour	0.46 ± 0.18	0.18 ± 0.1	<0.004
Light cycle phase duration (s) (hh:mm:ss)	5,789 ± 1,459	1,643 ± 1,188	<4.6 × 10^−5^
	01:36:29 ± 00:24:19	00:27:23 ± 00:19:48	
Number light cycle inactive states	5.8 ± 1.5	2.6 ± 1.2	<0.005
Light cycle inactive states per hour	0.55 ± 0.12	0.23 ± 0.1	<0.0002
Light cycle inactive state duration (min)	105 ± 28.6	235.1 ± 97.7	<0.002

**Figure 4:**
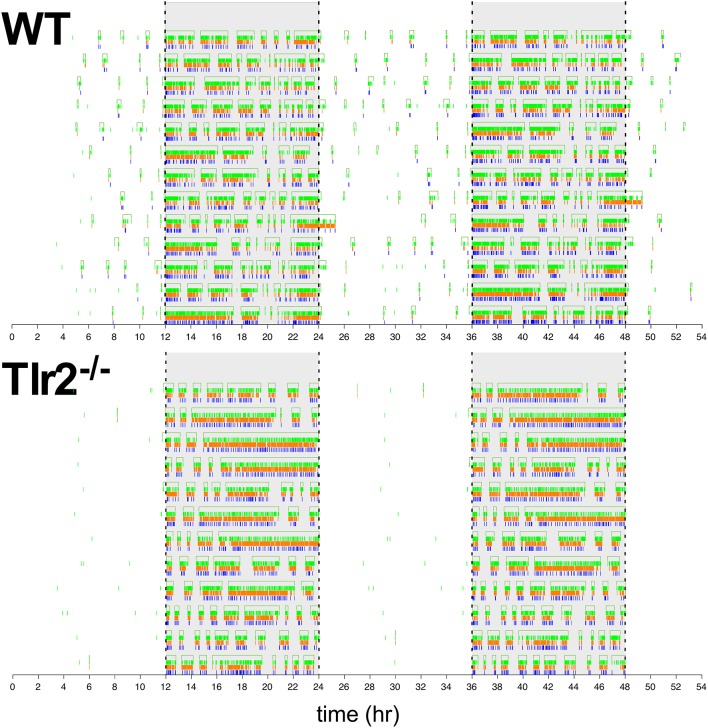
Dual plot actogram demonstrating dark cycle active state consolidation and light cycle inactive state consolidation of a representative Tlr2^−/−^ mouse in comparison to a representative WT mouse. X axis depicts military time (lights off at 12:00 and 36:00, lights on at 24:00 and 48:00) in hours. Y axis depicts experimental day. Note that the data shown in the last half of one daily trace is the first half of data shown in the following day (except for the last day of collection). Drinking, feeding, and movement events are depicted by blue, orange, and green hash lines, respectively. The empty rectangles at the top of tracings for each day represent duration of calculated active states. Dashed vertical lines show lighting transitions, gray background depicts dark cycle.

**Figure 5:**
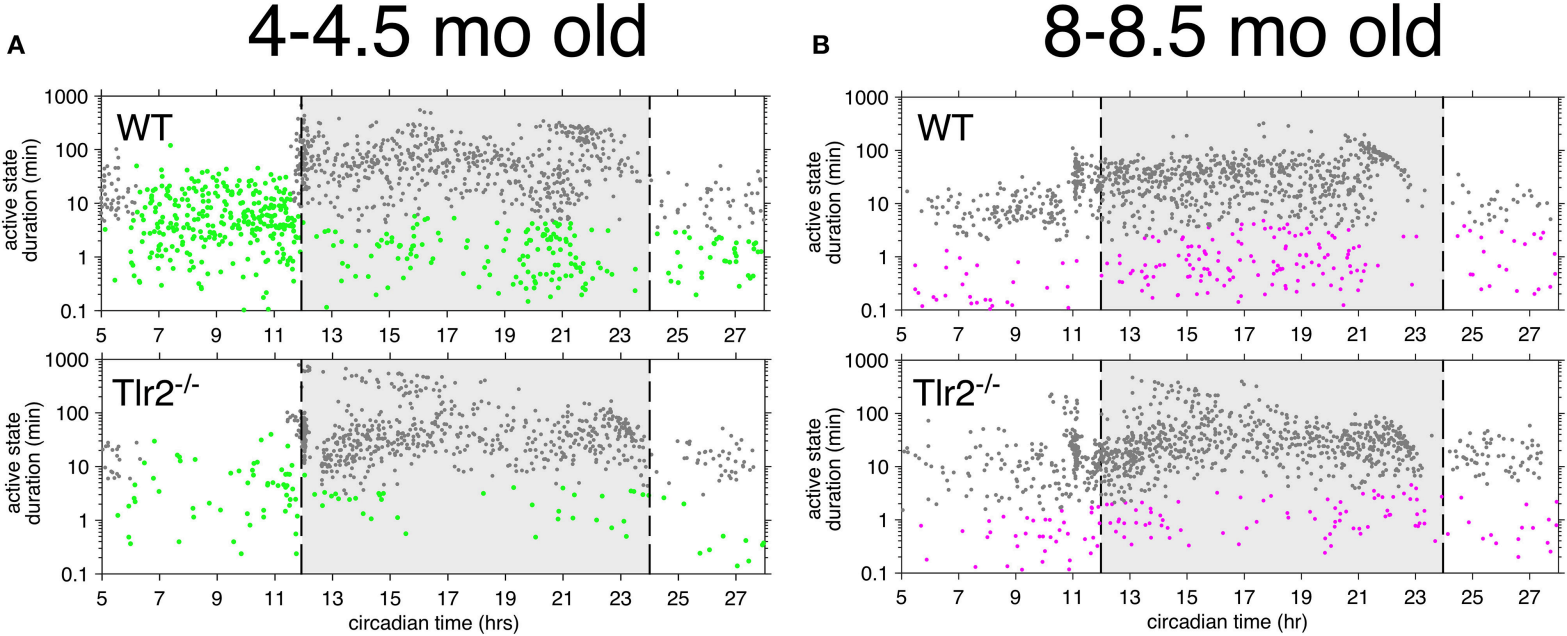
Tlr2^−/−^ mice have fewer short duration active states throughout the circadian day **(A)**. 4–4.5 months old mice. Each point represents an active state from a WT (top left) or Tlr2^−/−^ (bottom left) mouse within the cohort over the total experimental duration. x axis depicts active state onset time, y axis depicts active state duration (in log). Dashed lines depict onset (left) and offset (right) of the dark cycle, which is further highlighted in light gray. Active states highlighted in green depict regions where states with these specific onset/duration properties are statistically overrepresented in wildtype mice and underrepresented in Tlr2^−/−^ mice. Note that for 4–4.5 months old mice, these states include shorter duration active states throughout the circadian day, as well as all duration active states for the last half of the light cycle **(B)**. 8–8.5 months old mice. Each point represents an active state from a WT (top right) or Tlr2^−/−^ (bottom right) mouse. Plot annotations otherwise per **(A)**. Active states highlighted in violet depict regions where states with these specific onset/duration properties are statistically overrepresented in wildtype mice and statistically underrepresented in Tlr2^−/−^ mice. For 8–8.5 months old mice, these states include shorter duration active states throughout the circadian day.

This Tlr2^−/−^ light cycle phenotype is well illustrated in Figure 4, which shows a dual plot actogram for a representative 4–4.5 months old WT and Tlr2^−/−^ mouse over 16 days. Note that the Tlr2^−/−^ mouse demonstrated decreased light cycle activity, and greater active state consolidation over this time period compared to WT. These findings extend to all the mice within the WT and Tlr2^−/−^ cohorts (Figure 5A). For all mice within a cohort across all experiment days, Figure 5A depicts active state onset on the x axis and active state duration on the y axis and clearly demonstrates that Tlr2^−/−^ mice express fewer active states during the light cycle compared to WT. This plot also demonstrates that short duration active states occurring throughout the circadian dark cycle are statistically underrepresented in Tlr2^−/−^ mice.

### 8–8.5 months old Tlr2^−/−^ mice demonstrate fewer differences in active and inactive states

There were no genotypic differences between 8 and 8.5 months old WT and Tlr2^−/−^ mice in overall active/inactive state properties including (1) number of daily active states, (2) active state onset rates, (3) active phase durations, (4) number of daily inactive states, (5) inactive state onset rates, (6) inactive phase duration, or (7) inactive state duration. Similarly, there were no genotypic differences between 8 and 8.5 months old WT and Tlr2^−/−^ mice in light cycle active/inactive state properties including (1) number of light cycle active states, (2) light cycle active state onset rates, (3) light cycle active state duration, (4) number of light cycle inactive states, (5) light cycle inactive state onset rates, (6) light cycle inactive state duration, or (7) light cycle feeding, drinking, and movement. All of these metrics showed significant phenotypic differences between 4 and 4.5 months old WT and Tlr2^−/−^ mice.

However, 8–8.5 mo old Tlr2^−/−^ mice continue to demonstrate a statistically significant underrepresentation in short duration active states occurring during the dark cycle (Figure 5B), with this finding further extending to short duration active states within the light cycle. However, as apparent from comparison of similar time epochs between Figures 5A,B, this phenotype is less pronounced in the older mouse cohort.

### Tlr2^−/−^ mice show less variability in circadian patterns of movement, feeding, and drinking compared to WT

**Figure 6:**
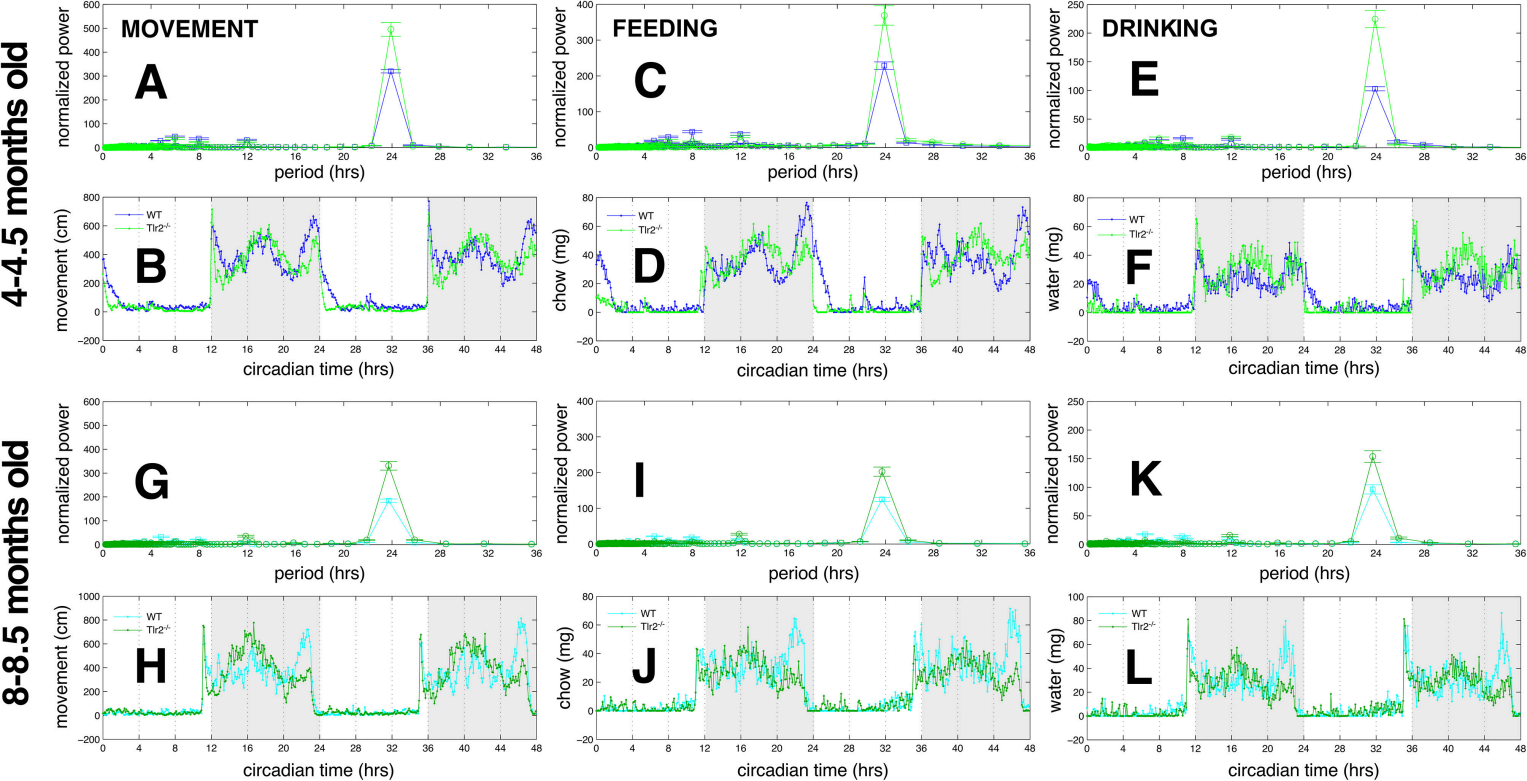
Tlr2^−/−^ mice have stronger 24-h periodicities compared to WT mice **(A)**. Lomb-Scargle periodogram for movement, 4–4.5 months old mice. WT periodogram in blue, Tlr2^−/−^ periodogram in green **(B)**. Observed movement for WT (blue) and Tlr2^−/−^ (green) cohorts, 4–4.5 months old mice. Thin line with points depicts the mean values for each cohort **(C)**. Lomb-Scargle periodogram for feeding. 4–4.5 months old mice. WT in blue, Tlr2^−/−^ in green **(D)**. Observed feeding for WT (blue) and Tlr2^−/−^ (green) cohorts. 4–4.5 months old mice. **(E)** Lomb-Scargle periodogram for drinking. 4–4.5 months old mice. WT in blue, Tlr2^−/−^ in green **(F)**. Observed drinking for WT (blue) and Tlr2^−/−^ (green) cohorts. 4–4.5 months old mice. **(G)** Lomb-Scargle periodogram for movement, 8–8.5 months old mice. WT periodogram in cyan, Tlr2^−/−^ periodogram in dark green **(H)**. Observed movement for WT (cyan) and Tlr2^−/−^ (dark green) cohorts. 8–8.5 month old mice. Thin line with points depicts the mean values for each cohort **(I)**. Lomb-Scargle periodogram for feeding. 8–8.5 months old mice. WT in cyan, Tlr2^−/−^ in dark green **(J)**. Observed feeding for WT (cyan) and Tlr2^−/−^ (dark green) cohorts. 8–8.5 months old mice **(K)**. Lomb-Scargle periodogram for drinking. 8–8.5 months old mice. WT in cyan, Tlr2^−/−^ in dark green **(L)**. Observed drinking for WT (cyan) and Tlr2^−/−^ (dark green) cohorts. 8–8.5 months old mice. For **(A**,**C**,**E**,**G**,**I**,**K)** error bars are ±1 standard deviation. No significant periodicities of longer than 24 h obtained for any behaviors.

We performed Lomb-Scargle analysis of movement, feeding, and drinking time series (from 4–4.5 to 8–8.5 months old WT and Tlr2^−/−^ mice) to assess periodicity of these behaviors. In both 4–4.5 months old mice and 8–8.5 months old mice, normalized power of the 24-h spectral component is significantly greater in Tlr2^−/−^ mice compared to WTs for movement, feeding, and drinking (Figure 6). The decrease in amplitudes of these 24-h spectral components (as well as 12-h and 8-h ultrudian spectral components) between 4–4.5 and 8–8.5 months old mice suggests that the older mice have greater variability in minute-to-minute and day-to-day performance of movement, feeding, and drinking compared with younger mice, and is consistent with our prior observations showing age-related decreases in the amplitude of these same spectral components in older C57BL/6 and BALB mice (unpublished data). Lomb-Scargle analysis suggests that Tlr2^−/−^ mice have less variability in movement, feeding, and drinking behaviors compared to WT mice, regardless of age.

### Tlr2^−/−^ mice have lower body weights than WT cohorts, but otherwise show no genotypic differences in metabolic parameters

WT mice were significantly heavier (25.2 ± 0.8 g WT; 23.1 ± 1.1 g Tlr2^−/−^; *p* < 0.0008 at 4–4.5 months; 29.0 ± 0.9 g WT; 26.7 ± 1.7 g Tlr2^−/−^; *p* < 0.004 at 8–8.5 months), and had a greater total tissue mass (TTM; 21.3 ± 0.8 g WT, 19.8 ± 1.0 g Tlr2^−/−^; *p* < 0.006 at 4–4.5 months; 25.4 ± 0.85 g WT, 23.3 ± 1.5 g Tlr2^−/−^; *p* < 0.005 at 8–8.5 months) than Tlr2^−/−^ mice. No significant differences in BMD, BMC, BArea, TArea, RST, or % adiposity were noted at either 4–4.5 or 8–8.5 months. We noted no significant differences in the metabolic parameters of $\dot{\text{V}}$O_2_, O_2_out, DO_2_, $\dot{\text{V}}$CO_2_, CO_2_out, DCO_2_, and heat either at rest (basal status) or with moderate activity.

### Phenotypes with no difference between WT and Tlr2^−/−^ cohorts in both 4-4.5 and 8-8.5 months old mouse cohorts

Regarding home cage phenotypes, we noted no significant differences in overall food and water consumption, and overall daily movement. There were no differences in the 24 h behavioral time budgets for inactivity, feeding, drinking, locomotor, and nonlocomotor movements. Similarly, there were no differences in active state time budgets examining percentage of time devoted to feeding, drinking, locomotion, and nonlocomotor movements, nor were there differences in the probabilities of specific behaviors occurring after start of new active states or preceding the finish of ongoing active states. We also found no significant differences in the dark cycle (4–4.5 months old) or dark and light cycle (8–8.5 months old) patterns of feeding, drinking, or movement bouts, including no differences in hourly bout onset rates, bout probabilities, bout speed/intensities, bout durations, or per-bout intake/movement. Finally, we assessed anxiety-related behaviors by elevated zero maze and open field assays (Supplemental Methods), and found that Tlr2^−/−^ mice demonstrated increased anxiety related behaviors (increased thigmotaxis and decreased entries into open field center; Supplemental Figure 1) compared to WT mice. No genotypic differences in zone transitions or time spent within either closed or open elevated zero maze arms were appreciated.

## Discussion

We provide the first description of Tlr2^−/−^ mouse behavior in a home cage environment assessed over an extended period of time. Most notably, we find that loss of Tlr2 function in 4–4.5 months old mice leads to a marked consolidation of both mouse active and inactive states, as determined by active/inactive state durations, onset frequencies, and a marked paucity of all behaviors occurring during the circadian light cycle. As a result of these changes, we also note that Tlr2^−/−^ mice demonstrate stronger day-to-day periodicity of movement, feeding, and drinking behaviors as revealed by Lomb-Scargle analysis. Of note, nearly all of these genotypic differences were no longer observed upon repeat examination of these same mouse cohorts at age 8–8.5 months, although there was still evidence of active/inactive state consolidation in older Tlr2^−/−^ mice.

Mechanisms underlying the loss of several of these phenotypes in older Tlr2 deficient mice are not currently understood. Our previous data demonstrate age-associated hypothalamic and cerebellar expression of multiple immune proteins (normally not present in young adult animals) that signal through similar mechanisms (Bonasera et al., 2016). Signaling through these aberrantly expressed molecular systems may offset the effects of Tlr2 loss. Further analysis of mice deficient in these specific immune proteins will offer insight regarding their contributions to active state regulation.

Behavioral phenotypes arising from Tlr2 loss remain unclear due to the limited scope of available data. Multiple studies suggest that Tlr2 functional loss is accompanied by altered energy balance and metabolism. For example, baseline hyperphagia accompanied by obesity and increased adiposity (on both regular and high fat diets), poor glucose tolerance, and increased respiratory ratio (RER) has been described in 4- and 7-months old Tlr2^tm1Aki^ constitutive knockout mice bred to a C57BL/6 background (Shechter et al., 2013). This same study also noted increased light cycle (and unchanged dark cycle) activity in 12 months old Tlr2 knockout compared to wildtype controls. By contrast, mice with Tlr2 lesions derived from a different founder strain (Tlr2^tm1Kir^) demonstrated hyperphagia, enhanced glucose tolerance, decreased insulin resistance and decreased RER on a high fat diet compared to controls (Ehses et al., 2010). Similar resistance to the metabolic syndrome phenotype was noted in 3+ months old Tlr2^tm1Aki^ mice bred to a C57BL6/Hsd background and receiving a high fat diet (Himes and Smith, 2010). Three months old mice with constitutive knockout of both Tlr2 and Tlr4 (bred to a C3H/HeJ background) maintained on a high fat diet for 8 weeks were noted to have lower body weights, enhanced glucose tolerance, and increased dark cycle locomotor activity compared to control mice kept on the same high fat diet (Sartorius et al., 2012). Our study finds no evidence of hyperphagia, obesity, or increased adiposity in Tlr2^−/−^ mice fed a regular diet compared to C57BL/6 controls; in fact, Tlr2^−/−^ mice were significantly lighter at 4–4.5 and 8–8.5 months time points compared to WT. These results differ from data presented by Shechter et al. (2013, their Figure 1B). We also did not observe any differences in metabolism between WT and Tlr2^−/−^ mice. There are no obvious factors to explain these discrepancies, which may be secondary to differences in mouse breeding strategy (heterozygous vs. homozygous pairings), housing (group vs. individual), diet (manufacturer/composition), or handling across different rodent colonies. Differences in how the two studies dispensed food (powdered *vs* pelleted chow) may also account for discrepancies in food intake.

Tlr2 functional loss has also been linked to sleep performance. EEG evaluation of sleep architecture revealed increased consolidation of both awake and sleeping states in Tlr2/Tlr4 double mutant mice, demonstrating significantly less NREM sleep during the dark cycle while simultaneously showing greater REM sleep during the light cycle compared to wildtype cohorts (Sartorius et al., 2012). Our observed phenotypes of dark cycle active state consolidation and light cycle inactive state consolidation are consistent with the sleep phenotypes reported by Sartorius and colleagues. Finally, there is evidence that Tlr2^tm1Aki^ mice have decreased anxiety-related behaviors (increased open field locomotion with decreased thigmotaxis, increased open arm dwell time on an elevated plus maze, decreased marble burying), impaired social behaviors (less recognition in a three-chamber social novelty assay, fewer contacts in a reciprocal social interaction test), increased aggression (more attacks in a cotton bud biting test), impaired sensorimotor gating (decreased prepulse inhibition of an otherwise normal acoustic startle response), and impaired cognition (diminished freezing to aversive context, diminished novelty seeking, poor performance in a Barnes maze task) compared to wildtype cohorts (Park et al., 2015). We noted increased anxiety-related behaviors as assessed by open field and elevated zero maze. Similar factors as discussed previously (for differences in body weight) may explain these discrepancies; we further note genetic differences between control C57BL/6J strains (Jackson vs. Daehan BioLink Inc.).

Given known expression of Tlr2 in LH orexinergic signaling pathways, we suspect that the behavioral impact of Tlr2 loss arises from defects in LH development, organization, or signaling. However, it is possible that loss of Tlr2 in peripheral cell types may also influence behavior. In particular, loss of Tlr2 expression in gut lymphoid cells may lead to variations in microbiota, which could influence behavior through multiple mechanisms including exposure to bacterial peptides, and/or release of hormones, neurotransmitters, or toxins (Carabotti et al., 2015 for review). Examination of differences in brain/gut axis between wildtype and Tlr2^−/−^ mice are beyond the scope of our study, but may constitute a future research direction.

Tlr2 signaling occurs when either Tlr1/Tlr2 heterodimers (in concert with Cd14) detect triacylated lipopeptides (Jin et al., 2007), or when Tlr2/Tlr6 heterodimers (in concert with Cd36/Cd14) detect diacylated lipopeptides (Kang et al., 2009). Tlr2 signaling in these contexts are early events promoting inflammation (Takeuchi and Akira, 2010) and sickness behavior (Jin et al., 2016). Active state regulation requires orexinergic/MCH neuronal signaling; it is therefore possible that behaviors associated with Tlr2 loss result from altered Tlr2 signaling in either orexin or MCH neurons, or interneuronal networks immediately upstream or downstream of these centers (Ohno and Sakurai, 2008; Apergis-Schoute et al., 2015). Orexinergic neurons strongly express both Tlr2 transcripts and protein (Dalal et al., 2013), and are thus potential loci where integration of animal health status and animal alertness/activity may occur. Tlr2 expressed by orexinergic neurons is thus particularly well placed to integrate animal health status and animal activity, since orexinergic neurons receive excitatory inputs from multiple CNS regions signaling potential life-threatening conditions (e.g., posterior hypothalamic nucleus (Yoshida et al., 2006; thermoregulation); ventrolateral preoptic nucleus (Sakurai et al., 2005; Yoshida et al., 2006; overall arousal state); bed nucleus of stria terminalis (Sakurai et al., 2005; anxiogenic states); amygdalar CRF projections (Winsky-Sommerer et al., 2004; stress responses); nucleus accumbens (Kirouac and Ganguly, 1995; Sano and Yokoi, 2007; reward); locus coeruleus and dorsal raphe nuclei (Pickel et al., 1974; Tabuchi et al., 2013; arousal states). Further, sickness behaviors have been directly linked to suppression of orexinergic signaling (Grossberg et al., 2011). Organization of CNS systems converging onto MCH neurons is not well-understood at this time; however, MCH neuronal activity can by modulated by neurotransmitters characteristic of the above systems, including glutamate, GABA, norepinephrine, and serotonin (Guyon et al., 2009). Our data demonstrating reduced activity fragmentation in mice lacking Tlr2 suggest that under conditions of increased Tlr2 activation fragmentation of activity, decreased active state duration, and reduced time spent in social contact with conspecifics may be observed. This response is an important aspect of current sickness behavior frameworks (Shakhar and Shakhar, 2015), providing a mechanistic connection for how individuals experiencing illness show strong tendencies to decrease movement and feeding behaviors.

In summary, we describe a previously unappreciated role of the pattern recognition receptor Tlr2 in regulating active state consolidation throughout the circadian day. As mentioned in the introduction, understanding mechanisms controlling active/inactive state transitions are highly relevant to understanding sleep/wake cycle regulation. Our data suggest that interventions to block Tlr2 signaling may be novel and promising avenues for treatment of insomnia and related sleep disturbances. Further research to identify how different pattern recognition receptors influence the orexinergic/MCH neuronal “switch,” and how signaling from these molecules ultimately affects neuronal architecture and function are critical to close current knowledge deficits.

## Ethics statement

This study was carried out in accordance with both institutional and federal guidelines regarding the use of laboratory animals. The protocol was approved by the University of Nebraska Medical Center Institutional Animal Care and Use Committee.

## Author contributions

ND: study conception, data collection, data analysis, manuscript preparation. TC: data collection, data analysis, manuscript preparation. SB: data analysis, manuscript preparation.

### Conflict of interest statement

SB is an uncompensated, *ad hoc* consultant to Mousera Inc., a startup company focused on high-throughput rodent behavioral monitoring technology. The other authors declare that the research was conducted in the absence of any commercial or financial relationships that could be construed as a potential conflict of interest.

## Supplementary material

Supplementary Figure 1 4–4.5 months old Tlr2^−/−^ mice show decreased locomotion and increased thigmotaxis in an open field assay of exploratory behavior. Leftmost plot depicts open field total distance. White bars depict WT (n = 8), grey bars depict Tlr2^−/−^ (n = 8). Error bars are ± 1 standard deviation. ^**^*p* < 0.01. Rightmost plot depicts open field center dwell time (left pair of bars, with corresponding left vertical axis) and number of center crossings (right pair of bars, with corresponding right vertical axis). Annotations per above. ^*^*p* < 0.05.

Supplementary Table 1 Tlr2^−/−^ and WT behavioral metrics. Column 1 lists the abbreviation for each behavior, Column 2 lists the overall behavioral assay class, Column 3 provides a brief description of the behavior, Column 4 is an index. Columns 5 and Column 6 list mean values for each behavior, control and Tlr2^−/−^, respectively. Columns 7–22 list results for each test by individual mouse. Control mice are listed in columns 7–14; Tlr2^−/−^ mice are listed in columns 15–22. Columns 23, 25, and 27 provide unadjusted *p*-values calculated by Mann–Whitney, Student's *t*-test, and fold change (B) methods. Columns 24 and 26 provide *p* values adjusted by false discovery rate (FDR) for Mann-Whitney (column 41) and Student's *t*-test (column 43). Column 28 lists the behavioral fold change.

## Abbreviations

Tlr2: toll-like receptor 2
LH: lateral hypothalamus
MCH: melanin concentrating hormone
DEXA: dual energy X-ray absorptiometry
BMD: bone mass density
BMC: bone mineral content
BArea: bone area
TArea: tissue area
R_*ST*_: ratio of soft tissue attenuation
TTM: total tissue mass
$\dot{\text{V}}$O_2_: maximum oxygen uptake
DO_2_: global oxygen delivery
O_2_out: oxygen output
$\dot{\text{V}}$CO_2_: maximum CO_2_ production
DCO_2_: global CO_2_ removal
CO_2_out: CO_2_ output
FDR: false discovery rate
WT: wildtype.
