# Supplementary figures and images for: Toll-Like Receptor 2 Is a Regulator of Circadian Active and Inactive State Consolidation in C57BL/6 Mice
Source: Front Aging Neurosci. 2017 Jul 14;9:219. doi: 10.3389/fnagi.2017.00219 (PMC5510442; doi:10.3389/fnagi.2017.00219)

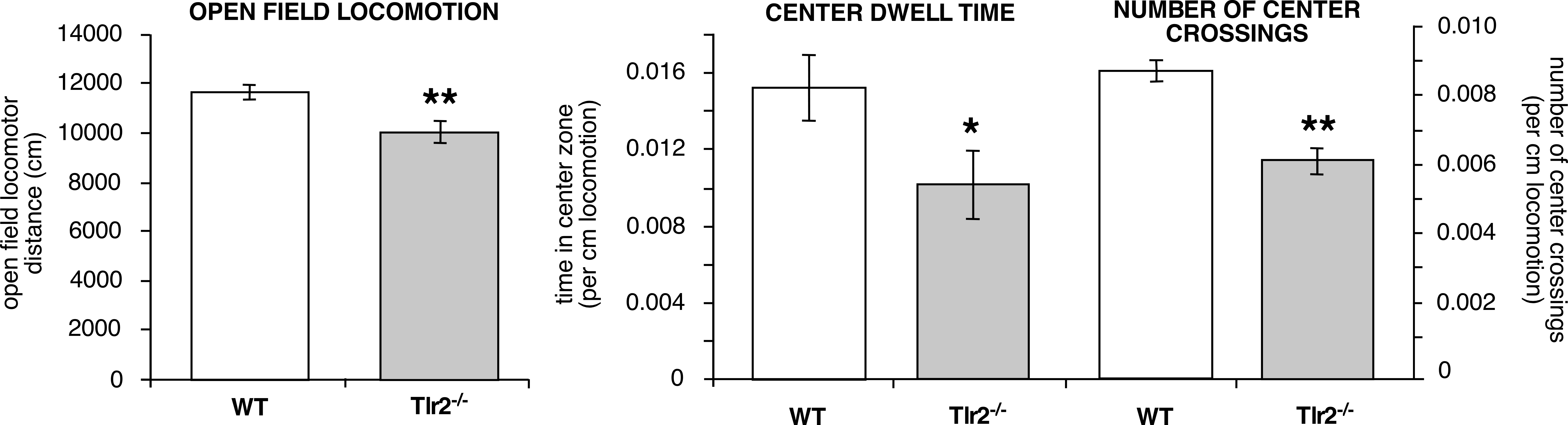

Supplement: Supplementary Figure 1 — 4–4.5 months old Tlr2−/− mice show decreased locomotion and increased thigmotaxis in an open field assay of exploratory behavior. Leftmost plot depicts open field total distance. White bars depict WT (n = 8), grey bars depict Tlr2−/− (n = 8). Error bars are ± 1 standard deviation. **p < 0.01. Rightmost plot depicts open field center dwell time (left pair of bars, with corresponding left vertical axis) and number of center crossings (right pair of bars, with corresponding right vertical axis). Annotations per above. *p < 0.05. [file Image1.PNG]
