# Supplementary material for: Toll-Like Receptor 2 Is a Regulator of Circadian Active and Inactive State Consolidation in C57BL/6 Mice
Source: Front Aging Neurosci. 2017 Jul 14;9:219. doi: 10.3389/fnagi.2017.00219 (PMC5510442; doi:10.3389/fnagi.2017.00219)
Supplement: Supplementary file 3 [file DataSheet1.docx]

**SUPPLEMENTAL METHODS/RESULTS.**

*Open field assay*. Mice were placed with their head facing into the same corner of an open field arena (49 cm long × 49 cm wide × 15 cm high, walls and floor colored white, custom fabricated by California Model and Design, San Francisco, CA). Locomotor activity was recorded at 30 frames/s to .mp4 format from a camera mounted directly above the arena; video files were analyzed using EthoVision XT8 (Noldus, Leesburg VA) to measure locomotor distance, center dwell time, center crossings, and thigmotaxis. We defined our thigmotaxis zone as extending 7 cm from the arena walls; the remainder of the arena was defined as the center zone. Mice were tested between 11:00 and 15:00 under room lighting. Trial duration was 20 minutes. Arena was washed with dilute Clidox-S solution, rinsed with 70% ethanol, and dried between mouse trials. We compared total open field distance between WT and Tlr2^-/-^ cohorts using unpaired, two-sided Student t-tests. We compared center crossings and center dwell time (normalized to locomotor distance) using unpaired, two-sided Student t-tests. We performed Bonferroni adjustment of critical p value to account for 3 comparisons.

We note that 4-4.5 mo old Tlr2^-/-^ mice demonstrate both significantly reduced locomotion (Supplemental Figure 1, left panel) and increased thigmotaxis (Supplemental Figure 1, right panel) compared to wildtype mice. These data suggest that loss of Tlr2 function in C57BL/6 mice is associated with decreased exploratory behavior and increased anxiety-related behaviors.

*Elevated zero maze*. Mice were placed in the center of the zero maze closed path (34 cm inner diameter, 46 cm outer diameter, on four-braced legs 58 cm off the ground; 12 cm high walls for closed path, white Plexiglas, California Model and Design, San Francisco). Behavior was recorded at 30 frames/s to .mp4 format from a camera mounted directly above the arena. Video files were analyzed using EthoVision XT8 (Noldus) to determine locomotor distance within the arena, and assessed for zone crossings and zone dwell time by manual observation. Zone crossings were scored only when all four of the animal’s paws crossed the border between open and closed regions of the maze. Mice were tested between 11:00 and 15:00 under room lighting. Trial duration was 6 minutes. Arena was washed with dilute Clidox-S solution, rinsed with 70% ethanol, and dried between mouse trials. We compared total zero maze locomotor distance between WT and Tlr2^-/-^ cohorts using unpaired, two-sided Student t-tests. We compared zone crossings and zone dwell times (normalized to locomotor distance) using unpaired, two-sided Student t-tests. We performed Bonferroni adjustment of critical p value to account for 3 comparisons.

We note that 4-4.5 mo old Tlr2^-/-^ mice demonstrate significantly reduced locomotion on the elevated zero maze (data not shown). No genotypic differences were appreciated in zone transitions, time spent on open arm, or time spent on closed arm.
